# Spraying Brassinolide improves Sigma Broad tolerance in foxtail millet (*Setaria italica* L.) through modulation of antioxidant activity and photosynthetic capacity

**DOI:** 10.1038/s41598-017-11867-w

**Published:** 2017-09-11

**Authors:** Xiang-Yang Yuan, Li-Guang Zhang, Lei Huang, Hui-Jie Yang, Yan-Ting Zhong, Na Ning, Yin-Yuan Wen, Shu-Qi Dong, Xi-E Song, Hong-Fu Wang, Ping-Yi Guo

**Affiliations:** 1 Key Laboratory of Crop Chemical Regulation and Chemical Weed Control, Agronomy College, Shanxi Agricultural University, Taigu, 030801 P. R. China; 2 Institute of Crop Sciences, Shanxi Academy of Agricultural Sciences, Taiyuan, 030032 P. R. China; 3 College of Agronomy and Biotechnology, Southwest University, Chongqing, 400715 P. R. China; 4 College of Resources and Environmental Sciences, China Agricultural University, Beijing, 100094 P. R. China

## Abstract

To explore the role of Brassinolide (BR) in improving the tolerance of Sigma Broad in foxtail millet (*Setaria italica* L.), effects of 0.1 mg/L of BR foliar application 24 h before 3.37 g/ha of Sigma Broad treatment at five-leaf stage of foxtail millet on growth parameters, antioxidant enzymes, malondialdehyde (MDA), chlorophyll, net photosynthetic rate (*P*_N_), chlorophyll fluorescence and P_700_ parameters were studied 7 and 15 d after herbicide treatment, respectively. Results showed that Sigma Broad significantly decreased plant height, activities of superoxide dismutase (SOD), chlorophyll content, *P*_N_, PS II effective quantum yield (Y (II)), PS II electron transport rate (ETR (II)), photochemical quantum yield of PSI(Y (I)) and PS I electron transport rate ETR (I), but significantly increased MDA. Compared to herbicide treatment, BR dramatically increased plant height, activities of SOD, Y (II), ETR (II), Y (I) and ETR (I). This study showed BR pretreatment could improve the tolerance of Sigma Broad in foxtail millet through improving the activity of antioxidant enzymes, keeping electron transport smooth, and enhancing actual photochemical efficiency of PS II and PSI.

## Introduction

Foxtail millet (*Setaria italica* L.), a main annual gramineae cereal food crop with good nutritional value, was originated in Northern China. Because of its better adaptability to barren and arid lands than other crops, foxtail millet plays an important role in agricultural production in arid and semi-arid regions of the world. Due to its small diploid genome, short life cycle, self-pollination, small adult stature and prolific seed production, domesticated foxtail millet is being promoted as a novel model species for functional genomics of the grass crop, especially for study of C_4_ photosynthesis^1, 2^.

One of the important constraints to foxtail millet production is weed infestation. Weeds compete for nutrients, water, light and space with foxtail millet, and seriously impact its yield and quality. Compared to conventional manual control, chemical weed control is much more efficient and labor-saving, and is extensively used in rice, maize, wheat and soybean production.

Foxtail millet is relatively more sensitive to many herbicides than rice, maize, wheat and soybean. However, It was reported that 0.45 kg/ha of 10% monosulfuron wettable powder^3^ and 3000 to 6000 mL/ ha of 38% atrazine^4^ treated pre-emergence are efficient to control broad leaf weeds and without causing injury to foxtail millet. It was also concluded that 2.1 to 2.4 kg/ha of monosulfuron plus propazine 44% water dispersible granule could provide 89.1 to 91.8% reduction in weed plant numbers and about 85% inhibition of weed fresh weight 45 days after pre-emergence application in foxtail millet^5^. The results of Song *et al*.^6^ showed that 180 g/ha of Sumisoya mixed with 300 kg/ha of (NH_4_)_2_HPO_4_ applied pre-plant has good control effect on crabgrass (*Digitaria sanguinalis* L.), barnyard grass (*Echinochloa crusgalli* (L.) Beauv), purslane (*Portulaca oleracea* L.) and redroot pigweed (*Amaranthus retroflexus* L.) and are safe to foxtail millet.

Similar to pre-emergence herbicides, there are also a few post-emergence herbicides being safely used in foxtail millet. It was reported that use of about 56% MCPA-sodium wettable powder was safe to foxtail millet according to earlier observations and can lead to control of about 92% of broad leaved weed plants^7^. However, Song *et al*.^8^ showed that MCPA and 2,4-D butyl ester were unsafe for use in foxtail millet. Use of Metsulfuron-methyl also resulted in drastic decline in net photosynthetic rate of foxtail millet^9^. However, compared to the water control, the application of dicamba at 4-leaf stage did not significantly decrease the yield of foxtail millet^8^. Even so, use of these herbicides sometimes have poor weed control and can even produce phytotoxic effect on foxtail millet because of dosage, application time, and other technical factors.

Sigma Broad, a water-dispersible granule mixture of mesosulfuron-methyl and iodosulfuron-methyl sodium, is a highly selective post-emergence herbicide developed by Germany’s Bayer Crop Science Company for control of some broadleaf weeds and vast majority of gramineae (malignant) weeds, and it’s known to work well even in winter wheat fields^10^. Although, both foxtail millet and wheat belong to the same gramineae family; use of Sigma Broad at recommend doses results in significant phytotoxicity to foxtail millet^11^.

Plant growth regulators are often applied to agricultural crops to induce stress tolerance. Brassinolide (BR) is one of the first isolated brassinosteroids, which are steroidal plant hormones regulating plant growth and development, and has been known to improve the crop tolerance to many abiotic stress factors such as high and low temperatures^12, 13^, drought^14^, water deficit^15^, salinity^16, 17^, heavy metals^18, 19^, pesticides^20^ including fungicides, insecticides and herbicides^21–23^. Brassinolide can stimulate photosynthesis, CO_2_ fixation under dark conditions, and the activities of ribulose-1,5-bisphosphate carboxylase (RuBPCase) and phosphoenol-pyruvate carboxylase (PEPCase) in the leaves of crops^24, 25^. In addition, it can modify the antioxidant enzyme activities^19^, membrane lipid peroxidation, protein content, other endogenous phytohormones and gene expression^26^ to increase the tolerance of crop to stresses.

The present study was therefore carried out to (1) investigate the possible physiological mechanism of herbicide Sigma Broad causing damage to foxtail millet, (2) explore whether BR can improve the tolerance of Sigma Broad in foxtail millet, and (3) understand the underlying physiological mechanism of BR in improving the tolerance of Sigma Broad in foxtail millet.

## Results

### Growth parameters

Seven days after Sigma Broad treatment, plant height and leaf area of both Jingu 21 and Zhangza 5 were markedly reduced. The fresh mass of Zhangza 5 was also decreased significantly in the herbicide treatment, although there were no significant differences between the control and the herbicide treatment for Jingu 21. Fifteen days after Sigma Broad treatment, plant height, leaf area and fresh mass of Jingu 21 were decreased by 61.3%, 57.3% and 72.4% respectively, while those of Zhangza 5 were reduced by 55.1%, 64.1% and 69.7% respectively.

Compared to herbicide (Sigma Broad) treatment, spraying 0.1 mg/L BR significantly enhanced the plant height of both cultivars 15 days after treatment (DAT) (Table 1). Although there were no significant difference in leaf area and fresh mass between “BR + Sigma Broad” and “Sigma Broad” treatment for both cultivars, leaf area in “BR + Sigma Broad” treatment was improved by 38.7% for Jingu 21 and 13.8% for Zhangza 5 respectively, and fresh mass was improved by 44.3% for Jingu 21 and 20.9% for Zhangza 5 respectively (Table 1).

**Table 1:** Effect of BR application on growth parameters in two foxtail millet cultivars under Sigma Broad treatment.

cultivars	time	treatment	plant height (cm)	leaf area (cm^2^)	fresh mass (g)
Jingu 21	7 DAT	Control (Water)	10.73 ± 1.07 a	15.15 ± 1.37 a	1.15 ± 0.40 a
		Sigma Broad	8.17 ± 0.57 b	9.54 ± 1.42 b	0.74 ± 0.41 a
		BR + Sigma Broad	10.47 ± 0.15 a	9.73 ± 0.46 b	0.98 ± 0.62 a
	15 DAT	Control (Water)	18.50 ± 0.70 a	21.78 ± 3.06 a	2.65 ± 0.49 a
		Sigma Broad	7.17 ± 2.02 c	9.29 ± 0.26 b	0.73 ± 0.40 b
		BR + Sigma Broad	11.03 ± 0.75 b	12.89 ± 0.72 b	1.05 ± 0.59 b
Zhangza 5	7 DAT	Control (Water)	12.40 ± 0.36 a	20.53 ± 3.81 a	1.68 ± 0.37 a
		Sigma Broad	8.23 ± 0.15 b	9.39 ± 1.68 b	0.87 ± 0.45 b
		BR + Sigma Broad	9.00 ± 0.87 b	9.59 ± 0.43 b	0.91 ± 0.54 b
	15 DAT	Control (Water)	15.23 ± 1.50 a	27.70 ± 7.62 a	2.53 ± 0.69 a
		Sigma Broad	6.83 ± 0.29 c	9.80 ± 0.41 b	0.77 ± 0.45 b
		BR + Sigma Broad	9.97 ± 1.15 b	11.15 ± 0.99 b	0.93 ± 0.66 b

Data are the mean ± standard error (n = 3). Duncan’s multiple range test at 5% probability level was used to compare the mean values of the treatment effects in each cultivar. Same letters after data indicate non-significant differences between treatments (P < 0.05).

### Protective enzyme activities and MDA content

Generally, at 7 DAT, protective enzymes superoxide dismutase (SOD), peroxidase (POD) and catalase (CAT) in both cultivars increased due to herbicide treatment alone and the level of increase in these protective enzymes was different between the cultivars (Table 2). At 15 DAT, the tested three protective enzymes in herbicide treatment showed a decreasing trend compared to the control except CAT in Zhangza 5. However, SOD, POD and CAT in both cultivars showed higher value in “BR + Sigma Broad” treatment than in “Sigma Broad” treatment at 7 and 15 DAT, respectively (Table 2).

**Table 2:** Effect of BR application on protective enzyme activities and malondialdehyde content in leaves of two foxtail millet cultivars under Sigma Broad treatment.

cultivars	time	treatment	SOD U/g Fw)	POD (ΔD470/g Fw/min)	CAT (U/g Fw/min)	MDA (µmol/g Fw)
Jingu 21	7 DAT	Control (Water)	217.8 ± 48.1 c	89.0 ± 34.4 c	388.5 ± 68.9 b	27.6 ± 10.8 c
		Sigma Broad	303.2 ± 20.6 b	116.1 ± 17.0 b	460.8 ± 5.2 ab	87.8 ± 17.4 a
		BR + Sigma Broad	416.7 ± 39.7 a	140.0 ± 29.9 a	490.0 ± 19.8 a	68.2 ± 16.6 b
	15 DAT	Control (Water)	253.4 ± 85.8 ab	107.0 ± 31.0 ab	405.5 ± 81.3 b	30.1 ± 5.5 b
		Sigma Broad	160.8 ± 42.5 b	71.0 ± 21.4 b	375.1 ± 32.5 b	98.5 ± 16.2 a
		BR + Sigma Broad	314.2 ± 20.4 a	119.9 ± 51.9 a	513.3 ± 18.9 a	75.5 ± 8.4 a
Zhangza 5	7 DAT	Control (Water)	274.2 ± 38.2 c	100.6 ± 20.7 a	402.7 ± 103.6 b	24.0 ± 6.6 b
		Sigma Broad	388.9 ± 45.6 b	118.7 ± 15.1 a	507.3 ± 11.7 ab	75.6 ± 23.6 a
		BR + Sigma Broad	557.8 ± 66.3 a	140.2 ± 54.9 a	534.2 ± 14.6 a	60.6 ± 24.7 ab
	15 DAT	Control (Water)	294.3 ± 47.8 b	85.3 ± 29.0 a	420.4 ± 68.5 a	28.9 ± 8.1 b
		Sigma Broad	262.1 ± 64.0 b	79.7 ± 14.9 a	427.5 ± 5.0 a	93.5 ± 20.8 a
		BR + Sigma Broad	491.7 ± 47.7 a	113.8 ± 32.7 a	461.0 ± 38.3 a	73.2 ± 10.9 a

Data are the mean ± standard error (n = 3). Duncan’s multiple range test at 5% probability level was used to compare the mean values of the treatment effects in each cultivar. Same letters after data indicate non-significant differences between treatments (P < 0.05).

SOD superoxide dismutase, POD peroxidase, CAT catalase, MDA malondialdehyde.

As shown in Table 2, herbicide significantly increased MDA in both cultivars at 7 or 15 DAT. However, compared to “Sigma Broad” treatment, “BR + Sigma Broad” treatment decreased MDA by 22.4% and 23.4% in Jingu 21, and 19.9% and 21.8% in Zhangza 5, respectively. MDA in “Sigma Broad” treatment increased with the extension of the time. However, MDA in “BR + Sigma Broad” treatment 15 DAT was lower than “Sigma Broad” treatment 7 DAT.

### Net photosynthetic rate (PN) and pigments

At 7 DAT, herbicide significantly decreased *P*_N_ in both foxtail millet cultivars compared to the water control. However, compared to herbicide treatment, BR increased *P*_N_ by 27.6% and 31.6%, respectively. At 15 DAT, differences between “BR + Sigma Broad” and “Sigma Broad” treatment in Jingu 21 reached significant level (Table 3).

**Table 3:** Effect of BR application on photosynthetic pigment content and net photosynthetic rate in leaves of two foxtail millet cultivars under Sigma Broad treatment.

cultivars	time	treatment	Chlorophyll (mg/g)	Carotenoid (mg/g)	Chl a/b	*P*_N_ (µmol/m^2^/s)
Jingu 21	7 DAT	Control (Water)	13.53 ± 4.27 a	2.29 ± 0.66 a	3.85 ± 0.30 a	18.86 ± 3.40 a
		Sigma Broad	8.56 ± 2.89 a	1.56 ± 0.50 a	3.61 ± 0.31 a	10.59 ± 0.83 b
		BR + Sigma Broad	10.42 ± 2.15 a	1.83 ± 0.19 a	3.57 ± 0.31 a	13.52 ± 0.98 b
	15 DAT	Control (Water)	19.80 ± 3.18 a	3.23 ± 0.49 a	4.45 ± 0.03 a	17.12 ± 1.22 a
		Sigma Broad	10.27 ± 2.97 b	2.12 ± 0.20 a	5.41 ± 1.50 a	8.27 ± 1.32 c
		BR + Sigma Broad	11.75 ± 0.28 b	2.30 ± 0.78 a	4.04 ± 0.81 a	12.12 ± 1.55 b
Zhangza 5	7 DAT	Control (Water)	12.20 ± 1.26 a	2.02 ± 0.17 a	3.89 ± 0.63 a	17.28 ± 2.55 a
		Sigma Broad	4.91 ± 1.89 b	1.18 ± 0.60 a	2.57 ± 0.72 a	9.85 ± 0.89 b
		BR + Sigma Broad	6.87 ± 0.85 b	1.56 ± 0.56 a	2.85 ± 1.49 a	12.96 ± 1.96 b
	15 DAT	Control (Water)	15.69 ± 5.89 a	2.98 ± 0.66 a	4.92 ± 0.98 a	16.64 ± 1.93 a
		Sigma Broad	7.99 ± 0.70 a	1.88 ± 0.36 a	6.06 ± 1.93 a	7.82 ± 1.12 b
		BR + Sigma Broad	8.80 ± 1.19 a	1.99 ± 0.44 a	5.61 ± 2.09 a	11.05 ± 2.36 b

Data are the mean ± standard error (n = 3). Duncan’s multiple range test at 5% probability level was used to compare the mean values of the treatment effects in each cultivar. Same letters after data indicate non-significant differences between treatments (P < 0.05).

Chl a/b chlorophyll a/b, PN net photosynthetic rate.

Sigma Broad significantly reduced Chl (a + b) (Chl (a + b)) content in Zhangza 5 at 7 DAT and in Jingu 21 at 15 DAT (Table 3). But, carotenoid (Car) and chlorophyll a/b (Chl a/b) in both cultivars were not significantly changed by the herbicide. Even so, carotenoid in Jingu 21 and Zhangza 5 15 DAT was decreased by 33.9% and 39.4%, respectively. Compared to the control, chlorophyll a/b (Chl a/b) in both cultivars were declined by 6.4% and 33.9% in herbicide treatment 7 DAT, and improved by 21.6% and 23.2% 15 DAT, respectively. Although there were not significant differences between “BR + Sigma Broad” and “Sigma Broad” in Chl (a + b), carotenoid and Chl a/b in both cultivars 15 DAT, Chl (a + b) in Jingu 21 and Zhangza 5 was improved by 14.4% and 10.2%, carotenoid improved by 8.2% and 9.5%, and Chl a/b decreased by 25.3% and 7.4%, respectively (Table 3).

### Chlorophyll fluorescence and P700 parameters

To further investigate the effect of “Sigma Broad” and “BR + Sigma Broad” treatment on photosynthetic apparatus of foxtail millet, the chlorophyll fluorescence and P_700_ parameters which show information about the state of photosystem II (PSII) and photosystem I(PSI) were measured.

Table 4 showed that except PSII maximum quantum yield (F_v_/F_m_) of Zhangza 5 in herbicide treatment 15 DAT, “Sigma Broad” and “BR + Sigma Broad” treatment did not significantly affect F_v_/F_m_, the quantum yield of non-photochemical losses via non-regulated pathways of PSII (Y (NO)) and the quantum yield of regulated energy dissipation in PSII (Y (NPQ)) in both cultivars. Y(II) is the effective quantum yield of PSII. However, no matter 7 DAT or 15 DAT, Sigma Broad significantly decreased Y (II) and PS II electron transport rate (ETR (II)). Compared to “Sigma Broad”, “BR + Sigma Broad” significantly increased Y (II) and ETR (II) 15 DAT, though the differences between the treatments were not significant 7 DAT.

**Table 4:** Effect of BR application on chlorophyll fluorescence parameters in leaves of two foxtail millet cultivars under Sigma Broad treatment.

cultivars	time	treatment	Y (II)	ETR (II)	Y (NO)	Y (NPQ)	Fv/Fm	Y(I)	ETR(I)	Y(ND)	Y(NA)	Pm
Jingu 21	7 DAT	Control (Water)	0.080 a	33.7 a	0.345 a	0.574 a	0.694 a	0.189 a	79.2 a	0.718 a	0.094 a	0.508 a
		Sigma Broad	0.067 b	28.2 b	0.372 a	0.561 a	0.665 a	0.159 a	66.8 a	0.756 a	0.085 a	0.386 a
		BR + Sigma Broad	0.073 ab	30.7 ab	0.346 a	0.581 a	0.673 a	0.173 a	72.8 a	0.751 a	0.076 a	0.396 a
	15 DAT	Control (Water)	0.088 a	37.0 a	0.312 a	0.600 a	0.728 a	0.192 a	80.5 a	0.754 a	0.055 a	0.514 a
		Sigma Broad	0.052 b	22.0 c	0.310 a	0.637 a	0.718 a	0.107 c	44.9 c	0.812 a	0.081 a	0.390 b
		BR + Sigma Broad	0.071 a	30.0 b	0.281 a	0.648 a	0.726 a	0.145 b	60.9 b	0.827 a	0.028 a	0.436 ab
Zhangza 5	7 DAT	Control (Water)	0.084 a	35.4 a	0.333 a	0.583 a	0.725 a	0.193 a	81.0 a	0.739 a	0.068 a	0.520 a
		Sigma Broad	0.064 b	27.0 b	0.321 a	0.616 a	0.661 a	0.156 b	65.8 b	0.775 a	0.069 a	0.315 b
		BR + Sigma Broad	0.074 ab	31.3 ab	0.351 a	0.575 a	0.681 a	0.185 a	77.7 a	0.725 a	0.090 a	0.416 ab
	15 DAT	Control (Water)	0.116 a	48.6 a	0.287 a	0.598 a	0.742 a	0.243 a	102.2 a	0.712 a	0.058 a	0.452 a
		Sigma Broad	0.058 c	24.6 c	0.302 a	0.640 a	0.711 b	0.143 b	60.0 b	0.745 a	0.113 a	0.415 a
		BR + Sigma Broad	0.090 b	38.1 b	0.304 a	0.606 a	0.723 ab	0.199 ab	83.5 ab	0.772 a	0.035 a	0.440 a

Data are the mean ± standard error (n = 3). Duncan’s multiple range test at 5% probability level was used to compare the mean values of the treatment effects in each cultivar. Same letters after data indicate non-significant differences between treatments (P < 0.05).

*Y (II)* PS II effective quantum yield, *ETR (II)* PSII electron transport rate, *Y (NO)* quantum yield of non-regulated energy dissipation in PSII, *Y (NPQ)* quantum yield of regulated energy dissipation in PSII, *Fv/Fm* PSII maximum quantum yield, *Y(I)* PSI photochemical quantum yield, *ETR(I)* PSI electron transport rate, *Y(ND)* quantum yield of non-photochemical energy dissipation due to donor side limitation in PSI, *Y(NA)* quantum yield of nonphotochemical energy dissipation due to accepter side limitation in PSI, *Pm* maximal P700 change.

Except Maximal P_700_ change (P_m_) of Zhangza 5 in herbicide treatment 7 DAT and P_m_ of Jingu 21 in herbicide treatment 15 DAT, “Sigma Broad” and “BR + Sigma Broad” treatment also did not significantly affect P_m_, quantum yield of non-photochemical energy dissipation due to donor side limitation in PSI(Y (ND)) and quantum yield of nonphotochemical energy dissipation due to accepter side limitation in PSI(Y (NA)) in both cultivars. Y(I) is the effective quantum yield of PSI. For Jingu 21, Y (I) and PSIelectron transport rate (ETR (I)) was not declined significantly in “Sigma Broad” treatment compared to the control 7 DAT, respectively. It was not improved significantly in “BR + Sigma Broad” treatment compared with “Sigma Broad” treatment 7 DAT, respectively. However, there were significant differences of Y (I) and ETR (I) in Jingu 21 between each treatment 15 DAT (Table 4). For Zhangza 5, Y (I) and ETR (I) in “Sigma Broad” treatment was significantly lower than the control 7 DAT and 15 DAT, respectively. “BR + Sigma Broad” treatment improved Y (I) and ETR (I) by 39.2% and 39.3% compared to “Sigma Broad” treatment 15 DAT, respectively, though there were not significant differences.

## Discussion

Each herbicide has its own killing scope and any incorrect use of herbicide will cause damage to crops. In this study, 3.37 g/ha of Sigma Broad significantly decreased the plant height, leaf area and fresh mass of both Jingu 21 and Zhangza 5 at 15 DAT (Table 1), suggesting it is unsafe to foxtail millet. This supported the work of Huang *et al*.^11^. Many literature have reported that BR can alleviate the injury of abiotic stresses such as high and low temperatures^12, 13^, salinity^16, 17^, water deficit^15^ and heavy metals^18, 19^ on the crops or improve the crop tolerance to such stresses. Some also reported that BR alleviated the phytotoxicity of pesticides on crops^20–23^. However, different crops and herbicide combinations may have different responses to certain plant growth regulator such as BR treatment. Xia *et al*.^20^ reported that 24-epivrassinolide (EBR) treated one day before fluazifop-p-butyl and haloxyfop alleviated the depressions on cucumber seedlings. Spraying BR after herbicide treatment could also alleviate the phytotoxicity of glyphosate and 2,4-D butyl ester on the cotton^21^. Our previous research also showed only BR foliar application could increase the plant height and fresh biomass of Jingu 21 and Zhangza 5^27^. In this paper, spraying 0.1 mg/L of BR 24 h before Sigma Broad treatment on foxtail millet significantly improved the plant height compared to Sigma Broad treatment alone, and enhanced leaf area and fresh mass by more than 38% and 13%, respectively (Table 1). This supports the previous results and suggests that BR can improve Sigma Broad tolerance in foxtail millet to some degree.

When plants are subjected to stress, reactive oxygen species (ROS) are over produced^19^. These ROS have damaging effects on plant process. Enzymes such as SOD, POD and CAT provide protection to plant against the damaging effects of ROS. In this study, activities of SOD, POD and CAT increased 7 DAT in different degrees may be to balance the generation and degradation of ROS caused by herbicide. However, with time extended, antioxidative enzyme activities declined and could not relief serious damage by Sigma Broad, and MDA increased 15 DAT (Table 2). Some papers also reported this result that herbicides could induce oxidative stress and lead to lipid peroxidation in plants^11, 28–31^. Plant hormones play the important role in regulation of stress response. It was documented that BR modified the activities of protective enzymes to improve the tolerance of plants under stress conditions^14, 15, 19^. Similar to previous researches, activities of SOD, POD and CAT in “BR + Sigma Broad” treatment increased much more 7 DAT to alleviate the herbicide injury compared with “Sigma Broad” treatment. This was also supported by MDA level higher in “Sigma Broad” treatment than in “BR + Sigma Broad” treatment 7 DAT. Although activities of SOD, POD and CAT in “BR + Sigma Broad” treatment declined a little 15 DAT due to severe herbicide injury, MDA was lower than in “Sigma Broad” (Table 2). These results suggest that BR treatment 24 hours before herbicide spraying can improve Sigma Broad tolerance in foxtail millet to certain degree by regulating activities of the protective enzymes.

Photosynthesis contributes more than 90% of crop biomass^32^, and is the basis of plant growth and development. Improper application of Sigma or Sigma Broad could dramatically decrease *P*_N_ of plants^10, 33, 34^. It was also demonstrated in Table 3 that *P*_N_ of foxtail millet in “Sigma Broad” treatment was significantly lower than the control. The decrease in *P*_N_ may partly be attributed to declined chlorophyll content (Table 3). Some studies^13–15, 20^ show that exogenous application of BR can improve photosynthesis under normal and stress (including herbicide) conditions. The present study also revealed that BR pretreatment increased *P*_N_ of foxtail millet at least 27% compared to “Sigma Broad” treatment although the differences were not significant between some treatments. One of the reasons for the increased *P*_N_ in BR treatment may also be due to increased chlorophyll content (Table 3). Zhang *et al*.^15^, Fariduddin *et al*.^35^, and Yuan *et al*.^36^ also reported that BR increased chlorophyll content under stresses.

Chloroplasts of photosynthetic apparatus, PSII and PSI in thylakoid membranes are the most sensitive parts to environmental stresses. Besides the primary effect, the secondary effect of the herbicide such as inhibition of photosynthesis system can also lead to death of plant. Different herbicides had various effects on chlorophyll fluorescence parameters of cucumber^20^. F_v_/F_m_ reflects the potential maximum photosynthetic ability of PS II.Y (II) reflects the actual photochemical efficiency of PSII. Paraquat dramatically inhibited F_v_/F_m_, Y (II), photochemical quenching (qP) and the non-photochemical quenching (NPQ)^20^. Although fluazifop-p-buty and haloxyfop had no significant effect on Fv/F_m_, fluazifop-p-buty showed a considerable decrease in Y (II) and q_P_, but an appreciable increase in NPQ^20^. For haloxyfop, it didn’t affect Y (II) and qP in cucumber^20^. Not only activities of PSII, but also activities of PSI was inhibited by herbicides^10, 17, 37^. Y (NO) reflects the fraction of energy that is passively dissipated in the form of heat and fluorescence, and is the important index of photo-damage in PSII. It consists of NPQ due to photo-inactivation of PSII and constitutive thermal dissipation that is very stable despite environmental stresses. Y (NPQ) represents the fraction of energy dissipated in form of heat via the regulated non-photochemical quenching, and is the important index of photo-protection in PSII. In this study, 3.37 g/ha of Sigma Broad did not significantly affect Y (NO) and Y (NPQ) in both foxtail millet cultivars and Fv/Fm of Jingu 21, but significantly decreased F_v_/F_m_ of Zhangza 5. This may suggest that, differences exist between cultivars for the influence of herbicide on the photosynthetic apparatus in foxtail millet. It may also suggest that although heat dissipation, fluorescence dissipation and protective enzyme mechanisms exist to protect the plant against various stresses, it is beyond the regulating ability, the structures of PSII is seriously damaged, and the potential maximum photosynthetic ability is influenced in Sigma Broad stress. In addition, the absolute values of F_v_/F_m_ in all treatments were lower than 0.78, showing the seedlings were subjected to other stresses and photoinhibitory damage of PSII was produced. This may be due to the high air temperature (37.2–40.4 °C) during the treatment period.

Typical feature of PSI photoinhibition is the decline in maximum oxidation–reduction ability of PSI^10, 38^. P_m_ represents the quantity of efficient PS I complex. Y (NA) is the important index of photo-damage in PSI. Inactivation of the key enzymes and electronic accumulation of acceptor-side in PSI caused by the damage of *Calvin-Benson* circle after dark adaption can lead to the increase of Y (NA)^39^. Y (ND) is the important index of photo-protection in PSI. P_m_ was decreased by 8.19% and 24.02% 15 DAT in Zhangza 5 and Jingu 21 compared to the control, respectively (Table 4), showing Sigma Broad produced some damage to PSI.Y (NA) in “Sigma Broad” treatment was more than 47% higher than the control 15 DAT, suggesting the fraction of overall P_700_ that could not be oxidized was increased and inactivation of the key enzymes of *Calvin-Benson* circle might cause electronic accumulation of acceptor-side in PSI. Y (I) reflects the actual photochemical efficiency of PSI. Y (II), ETR (II), Y (I) and ETR (I) were significantly decreased in “Sigma Broad” treatment 7 DAT or 15 DAT (Table 4), showing the actual photochemical efficiency and electron transport efficiency of PSII and PSI were inhibited significantly. D_1_ protein in PSII reaction center is extremely easily damaged. To maintain the function of PSII reaction center, reproduction and degradation of D_1_ must be maintained in relative balance^37, 40^. Sigma Broad, one kind of acetolactate synthase (ALS) herbicide, inhibits the synthesis of branched chain amino acids and disturb the amino acids pool, which may be important to synthesis of D_1_ protein. In this research, ETR (II) was smaller than ETR (I) suggesting that the PSI-driven cyclic electron flow might be stimulated to protect PSI from Sigma Broad damage^37, 41^.

BR pre-spraying enhanced F_v_/F_m_, RuBPcase and PEPcase of soybean in drought stress^15^, enhanced F_v_/F_m_, Y (II), q_P_ and q_N_ of cucumber in Ca(NO_3_)_2_ stress^36^, and increased F_v_/F_m_, Y (II), q_P_, the maximum carboxylation rate of Rubisco (Vcmax), the maximum potential rate of electron transport contributed to Ribulose-1,5-bisphosphate (RuBP) (Jmax) of tomato in heat stress^42^. Xia *et al*.^20^ showed that BR pretreatment increased F_v_/F_m_, Y (II) and qP, but decreased NPQ in paraquat, fluazifop-p-buty and haloxyfop stress in different extent, respectively. Yuan *et al*.^36^ also revealed that BR relieved the damage of internal lamellae of the stromal thylakoids and chloroplast envelopes by protecting the photosynthetic membrane system from oxidative stress. In present study, “BR + Sigma Broad” significantly increased Y (II), ETR (II), F_v_/F_m_, Y (I), ETR (I) and P_m_ which were dramatically decreased by “Sigma Broad” 7 DAT or 15 DAT. These results are similar to the previous, suggesting that BR improves Sigma Broad tolerance in foxtail millet by regulating the systems of PS II and PS I. Y (NA) in “BR + Sigma Broad” treatment was more than 64% lower than “Sigma Broad” treatment 15 DAT (Table 4), suggesting BR may also improve the key enzymes of *Calvin-Benson* circle to reduce the herbicide damage to plant.

## Conclusion

In conclusion, 3.37 g/ha of Sigma Broad made significant damage to foxtail millet by inducing oxidative stress and lead to lipid peroxidation, reducing the chlorophyll content, damaging the system of PS II and PS I, and decreasing the photosynthetic rate. Spraying 0.1 mg/L of BR 24 h before Sigma Broad treatment could partially alleviate the negative effect and improve the tolerance of Sigma Broad in foxtail millet through improving the activity of antioxidant enzymes, decreasing lipid peroxidation, increasing the chlorophyll content, keeping electron transport smooth, enhancing actual photochemical efficiency of PS II and PS I, and increasing the photosynthetic rate.

## Materials and Methods

### Plant materials and experiment design

Experiments were conducted at Shanxi Agricultural University, Shanxi, China. Two foxtail millet cultivars, Zhangzagu 5 and Jingu 21, were used in this experiment. Zhangzagu 5 was supplied by Zhangjiakou Academy of Agricultural Sciences of Hebei Province, China. Jingu 21 was supplied by the Institute of Economic Crops, Shanxi Academy of Agricultural Sciences, China. BR wettable powder (0.01%) (*Chengdu New Sun Crop Science Co., Ltd*. Sichuan, China) and Sigma Broad water dispersible granule (3.6%) (*Bayer Crop Science Co., Ltd*. Hangzhou, China) were also used in this experiment.

The experimental design was a split-plot with three replications, with two foxtail millet cultivars in the main plots and chemical treatments (water, Sigma Broad and “BR + Sigma Broad”) in the sub-plots, and each replication contained three pots. Twenty seeds for each cultivar were planted in 1 cm depth equidistantly in a plastic pot with 15 cm diameter by15 cm height filled with a growth medium consisting of a 1: 2 mixture of sand and loam soil with moderate fertility. The pots were placed on the ground outside the laboratory and carefully watered. Seedlings were thinned to five plants per pot at three-leaf stage.

With regard to previous results^11^, BR with effective dosage of 0.1 mg/L was foliar applied at five-leaf stage, because this concentration had the best positive effect on the growth of foxtail millet. Sigma Broad at 3.37 g/ha effective dosage with 0.4% v/v alkyl ethyl sulfonate as adjuvant was sprayed 24 hours later according to Zhong *et al*.^27^. The reason for using this dosage was because it not only produced phytotoxicity to foxtail millet but also probably was alleviated by plant growth regulator. The application was made with a hand-held sprayer, calibrated to deliver 450 L/ha. Equal amount of water was sprayed as the control.

### Measurements

The penultimate leaf of foxtail millet seedlings were used for the following physiological and biochemical estimations. Samples were collected 7 and 15 days after the herbicide treatment (DAT), respectively.

#### Measurement of growth parameters

Plant height of foxtail millet seedlings was measured with ruler. Leaf area was calculated by the following equation: leaf area = 0.75 × leaf length × leaf width^43^. Leaf width is the widest part of the penultimate leaf. The fresh mass was weighed by one ten-thousandth analytical balance (*Mettler-Toledo, LLC*. Shanghai, China).

#### Determination of antioxidant enzymes activities

Fresh foxtail millet leaf (0.1 g) was weighed into an ice-cooled mortar, ground in an ice bath with 1.5 mL 0.1 mol/L phosphate buffer (PH 7.8), and centrifuged at 12000 × g for 15 min at 4 °C. The supernatant was extracted for activity of SOD, POD and CAT determination.

SOD activity was determined by the nitro blue tetrazolium method^44^. One unit of enzyme activity (U) was defined as the amount of enzyme required to inhibit 50% of the initial reduction of NBT under the light conditions. SOD activity was presented as the number of U per gram fresh weight (U/g FW).

POD activity was determined according to the guaiacol method described by Gao^44^ with some minor modifications. 5 μL of enzyme extract was added into 3 mL reaction liquid consisting of 3 mL of phosphate buffer (pH 6.0), 30% hydrogen peroxide (H_2_O_2_) and guaiacol (AR) for 3 min. POD activity was evaluated by the absorbance change at 470 nm per min.

CAT activity was determined by the ultraviolet absorption method^44^. One activity unit (U/g FW/min) is defined as the enzymatic amount reduced by 0.1 per min at ΔD_240_. For CAT activity determination, the reaction mixture consisted of 2.7 mL Tris-HCl (PH 7.0), 50 μL H_2_O_2_ (200 mmol/L) and 20 μL enzyme extract. The absorbance was measured at 240 nm and recorded every 30 s in 3 min.

#### Determination of MDA

MDA is the end product of lipid peroxidation which reflects the level of membrane damage. It was determined by the thiobarbituric acid (TBA) test according to Gao^44^. Leaf samples (0.1 g) were homogenized with 5 mL of 0.1% trichloroacetic acid (TCA), 5 mL of 0.5% TBA was added and mixed well in a glass test tube. The reaction mixture solution was boiled for 15 min, cooled quickly, and centrifuged for 15 min at 4000 × g to clarity precipitation. Absorbance was measured at 532 nm and 600 nm, respectively.

#### Determination of *P*_N_ and pigments

*P*_N_ of the penultimate leaf was measured by *CI-340* portable photosynthesis system (*CID Bio-Science, Inc*., USA) during 9:30 to 10:30. Photosynthetically active radiation (PAR) at leaf surface was about 1050 ± 100 μmol/m^2^/s, temperature in leaf chamber was 38.8 ± 1.6 °C, ambient CO_2_ concentration was 419.4 ± 10 μmol/mol.

Chl (a + b) and Car contents were determine following the method of ethanol extraction^44^ with some adjustments. The penultimate leaf was cut into small pieces, then well mixed. 0.1 g of samples were placed in 15 ml scale test tube along with 10 ml of 96% ethyl alcohol and then covered with rubber stopper and kept at dark cabinet for at least 24 h until the liquid changed to white. During the period, the tube was shaken 3 to 4 times. Chl (a + b) and Car concentrations were measured using a 722 ultraviolet-visible spectrophotometer (*Shanghai Metash Instruments Co., Ltd*) and absorbance was measured at 470 nm, 649 nm and 665 nm.

$$\begin{array}{c}{{\rm{C}}}_{{\rm{a}}}=13.95\times {{\rm{A}}}_{665}\,\mbox{--}\,6.88\times {{\rm{A}}}_{649}\\ {{\rm{C}}}_{{\rm{b}}}=24.96\times {A}_{649}\,\mbox{--}\,7.32\times {{\rm{A}}}_{665}\\ {{\rm{C}}}_{{\rm{Car}}}=1000\times {{\rm{A}}}_{470}\,\mbox{--}\,2.05\times {{\rm{C}}}_{{\rm{a}}}\,\mbox{--}\,114.8\times {{\rm{C}}}_{{\rm{b}}}/245\end{array}$$

$${\rm{Pigmentcontent}}\,(\mathrm{mg}/g\,{\rm{Fw}})={\rm{C}}\times {{\rm{V}}}_{{\rm{T}}}\times {n/F}_{{\rm{W}}}\times {\rm{1000}}$$

In equation, C is the pigment concentration (mg/L), F_W_ is fresh weight (g), V_T_ is total volume of the extraction (mL), and n is the dilution ratio.

#### Measurement of chlorophyll fluorescence and P_700_ parameters

Chlorophyll fluorescence and P_700_ parameters were performed as previously reported by Yuan *et al*.^10^ and simultaneously measured by *Dual-PAM-100* measurement system (*Germany WALZ company*), using the automated Induction and Recovery Curve routine provided by the *Dual PAM* software. Prior to measurements, treated seedlings were placed in darkroom for 30 min, and fluorescence induced curve (Slow Kinetics) was determined in “Fluo + P_700_ mode”. Then, the kinetics of chlorophyll fluorescence induction and P_700_ oxidation were recorded simultaneously by the instrument. Firstly, the initial fluorescence (F_0_) was established and subsequently F_m_ was determined by the Saturation Pulse method. Then, P_m_ was determined by application of a saturation pulses (SP) after far-red pre-illumination. At last, actinic illumination was started and SP was given every 20 s, with the same pulses serving for fluorescence and P_700_ analysis.

${{\rm{F}}}_{{\rm{v}}}/{{\rm{F}}}_{{\rm{m}}}$ was evaluated as ${{\rm{F}}}_{{\rm{v}}}/{{\rm{F}}}_{{\rm{m}}}=({{\rm{F}}}_{{\rm{m}}}\,\mbox{--}\,{{\rm{F}}}_{{\rm{0}}})/{{\rm{F}}}_{{\rm{m}}}$. Other PS II energy dissipation parameters were estimated by the Dual PAM software. Apparent electron transfer efficiency in PS II in light was calculated according to ETR (II) = PAR × 0.84 × 0.5 × Y (II), and was used to measure electron transfer of carbon fixation resulted from photochemical reactions. Three complementary quantum yields of energy conversion in PS II were calculated^45^: Y (II) was evaluated as $Y({\rm{{\rm I}}}{\rm{{\rm I}}})={{({\rm{F}}}_{{\rm{m}}}}^{^{\prime} }-{{\rm{F}}}_{{\rm{m}}})/{{{\rm{F}}}_{{\rm{m}}}}^{^{\prime} }$, the yield of non-photochemical losses via non-regulated pathways of PS II as ${\rm{Y}}({\rm{NO}})=1/({\rm{NPQ}}+1+{\rm{qL}}\times ({{\rm{F}}}_{{\rm{m}}}/{{\rm{F}}}_{{\rm{0}}}\,\mbox{--}\,{\rm{1}}))$, ${\rm{NPQ}}={({\rm{F}}}_{{\rm{m}}}/{{{\rm{F}}}_{{\rm{m}}}}^{^{\prime} }\,-\,1$, ${\rm{qL}}={\rm{qP}}\times {{{\rm{F}}}_{{\rm{0}}}}^{^{\prime} }/{\rm{F}}$, ${\rm{qP}}=({{{\rm{F}}}_{{\rm{m}}}}^{^{\prime} }\mbox{--}{\rm{F}})/({{{\rm{F}}}_{{\rm{m}}}}^{^{\prime} }\mbox{--}{{{\rm{F}}}_{{\rm{0}}}}^{^{\prime} })$, where quantum yield of regulated energy dissipation in PS II as ${\rm{Y}}({\rm{NPQ}})=$ $1\,\mbox{--}\,{\rm{Y}}({\rm{II}})\,\mbox{--}\,{\rm{Y}}({\rm{NO}})$.

P_700_ oxidation was monitored by absorbance changes in the near-infrared (830–875 nm)^46^. The maximal P_700_ signal observed upon full oxidation was denoted by P_m_. Y(NA), the quantum yield of non-photochemical energy dissipation due to acceptor-side limitation, was calculated according to: $Y(\mathrm{NA})={{(P}_{{\rm{m}}}}^{^{\prime} }-{{\rm{P}}}_{{\rm{m}}})/{{{\rm{P}}}_{{\rm{m}}}}^{^{\prime} }$. Photochemical quantum yield of PSIas Y (I) was estimated according to $Y(I)={{(P}_{{\rm{m}}}}^{^{\prime} }-{\rm{P}})/{{\rm{P}}}_{{\rm{m}}}$; Quantum yield of non-photochemical energy dissipation due to donor side limitation in PS I as Y(ND) was calculated by $Y(\mathrm{ND})=(P-{{\rm{P}}}_{0})/{{\rm{P}}}_{{\rm{m}}}$. The total value of three quantum yields was one: Y (I) + Y (ND) + Y (NA) = 1. The electron transfer efficiency of PS I as ETR (I) was provided by the *Dual PAM* software.

### Statistical analysis

Data were analyzed using the Data Processing System (*DPS 7.05*) program package according to one-factor randomized complete block design analysis of variance (*ANOVA*), and the *Duncan’s* multiple range test at 5% probability level was used to compare the mean values of the treatment effects in each cultivar. The values sharing the same letters are not significantly different at 5% level.
